# Letter to editor about acupuncture for rehabilitation after total knee arthroplasty: a systematic review and network meta-analysis

**DOI:** 10.1097/JS9.0000000000003578

**Published:** 2025-10-06

**Authors:** Xiao Han, Min Xia

**Affiliations:** a Department of Rehabilitation Medicine, Ningbo Beilun Third People’s Hospital, Ningbo, China; b Department of Acupuncture, Community Health Service Center of Jiaochuan Sub-district, Zhenhai District, Ningbo, China

*Dear Editor,*

We read with great interest the recently published network meta-analysis that evaluated the effectiveness of various acupuncture-related therapies on postoperative recovery following total knee arthroplasty (TKA)^[1]^. The authors should be commended for conducting a comprehensive synthesis of randomized controlled trials (RCTs) and for attempting to disentangle the comparative benefits of different acupuncture modalities. Given the rising global prevalence of knee osteoarthritis and the persistent challenges of pain and functional limitation after TKA, such work is both timely and valuable. We appreciate the authors’ contribution to this field. Nevertheless, we believe several issues merit further discussion to place these findings in an appropriate clinical and methodological context.

First, among the 28 RCTs included, 21 were conducted in mainland China or Taiwan. This reflects the cultural and clinical prominence of acupuncture in East Asia. However, acupuncture practices vary across regions, and patients’ acceptance as well as placebo responses may differ substantially depending on whether they are familiar with traditional Chinese medicine^[2]^. Such geographical clustering could limit the generalizability of the conclusions, particularly for Western health systems where acupuncture is less common and often used as a complementary therapy.

The results suggest that electroacupuncture may provide greater benefits in reducing pain and stiffness, with a standardized mean difference of approximately 0.58. While statistically significant, the clinical relevance of this effect remains uncertain. In the setting of postoperative rehabilitation, even modest improvements need to be weighed against practical considerations such as the availability of trained personnel, integration with physiotherapy, and patient preferences^[3]^. Future studies that report absolute changes in functional outcomes, for example, walking speed or quadriceps strength would better inform clinicians about the real-world implications of these findings.

Another concern is the limited reporting of safety outcomes. Although some minor adverse events were documented, such as local pain, dizziness, and nausea, systematic evaluation of more serious complications was lacking. This is particularly important, as patients recovering from TKA often have comorbidities, impaired coagulation, and reduced mobility, all of which could increase the risks associated with invasive procedures like acupuncture. We encourage future trials to incorporate structured follow-up to more rigorously assess safety in postoperative populations.

In summary, this network meta-analysis provides valuable insights into the comparative role of acupuncture therapies in TKA rehabilitation, with electroacupuncture showing particular promise. However, the evidence is largely confined to a single cultural setting, and issues of methodological heterogeneity and potential placebo effects warrant cautious interpretation. Future large-scale, multicenter RCTs with broader international representation, rigorous blinding, and standardized reporting are needed. Such studies will be critical to determine whether acupuncture can truly transcend cultural familiarity and trial context to improve postoperative outcomes across diverse patient populations worldwide. This article was created in accordance with the TITAN guidelines^[4]^.

## Data Availability

There are no data.
